# Editorial: Insights and advances in pediatric critical care

**DOI:** 10.3389/fped.2022.1057991

**Published:** 2022-10-31

**Authors:** Jhuma Sankar, Niranjan Kissoon

**Affiliations:** ^1^ Division of Pediatric Pulmonology and Critical Care, Department of Pediatrics, All India Institute of Medical Sciences, New Delhi, India; ^2^ Department of Pediatrics and Emergency Medicine, UBC & BC Children’s Hospital, UBC Vancouver, Canada

**Keywords:** insights and advancements, insights, pediatric critical care, pediatric intensive care, artificial intelligence, fluids, cardiac critical care

**Editorial on the Research Topic** Insights and advances in pediatric critical care By Sankar J, Kissoon N. (2022) Front. Pediatr. 10: 1057991. doi: 10.3389/fped.2022.1057991

Future progress in paediatric critical care (PCC) will be accelerated by harnessing the collective wisdom of our colleagues globally. It is for this reason that we invited participation in this collection of Frontiers on Insights in Pediatric Critical Care. In this collection we have put together a unique collection of contemporary research work encompassing varied critical care conditions and what the future holds for these conditions.

The year 2020 saw the dawn of another era in medicine with the arrival of SARS CoV-2. Sooman et al. in eight Swiss PICUs during the pandemic which had eight Pediatric Multisystem Inflammatory Syndrome- Temporally associated with SARS CoV-2 (PIMS-TS) patients observed higher workload, different organisational issues due to temporary regulations by the government. While some of the PICUs admitted PIMS-TS patients, others did not and this resulted in poor bed occupancy rates and skewed utilisation of resources. We learn from their experience that differences between PICUs within a country are substantial and distribution of resources should be addressed.

The disease profile in PCC has changed over time with fewer but severe infections, caring for children with complex surgical procedures, acute multisystem involvement, chronic co-morbidities and more severe conditions necessitating greater complexity of services (1, 2). As a result, we have graduated from early “rudimentary” support to complex therapies such as extracorporeal therapies in lung and heart problems, use of targeted therapies like mono clonal antibodies and use of biomarkers for individualised approach.

As survival rates improved, long term morbidities increased and survival of those with chronic illnesses improved. Pediatric palliative care (PC) is slowly emerging as a major component of supportive therapy in pediatric critical care and is recommended in life threatening illness and not only life limiting illness. In the article by Buang et al., the authors highlight the importance and principles of palliative care and provide the pyramid model framework of integrating PC into ICU.

While optimising ventilation takes care of the primary lung condition, optimising fluid, electrolyte and nutritional support and preventing infections are key to successful ventilation and survival. Appropriate fluid management in mechanically ventilated critically ill children remains an important challenge and in this issue Arrahmani and team with an aim to understand the current fluid therapy practices in mechanically ventilated children survey 107 intensivists with more than 10 years experience. About 75% administered restrictive fluids (80%), greater than 5% fluid overload% prompted diuretic administration and only about 50% of participants used lung ultrasound to identify pulmonary edema. Further studies addressing restrictive fluid strategies are the need of the hour.

Resuscitation in septic shock in 1991 revealed the need for generous fluid resuscitation to improve outcomes (3). Boluses of 20 mls/kg repeated several times became accepted practice and was the basis of the Early Goal Directed Therapy (4, 5), and septic shock guidelines including that for paediatrics (6). However, PCC is practiced in environments endowed with high technology and at the other extreme austere settings. The finding that bolus fluid can be harmful in resource poor settings (FEAST) (7) has led to investigations of less aggressive fluid resuscitation (SQUEEZE) (8). Fluid bolus administration requires careful monitoring for features of fluid overload and use of point-of-care ultrasonography (POCUS) to aid in clinical decision making in determining fluid responsiveness and cardiac output. Ultrasonography has evolved as a useful tool in the PICU and Emergency Department (ED). Burton et al. in the present series describe the current state, challenges and future direction of POCUS in the PICU and highlight the importance of training, standardisation of usage and competency while using POCUS.

Determining fluid responsiveness using only clinical parameters has been found to be unreliable in the ED. Awadhare P et al. in 40 patients with shock evaluated the utility of a relatively new non-invasive monitoring device (ICON monitor) in a before and after bolus study. A 15% increase in stroke volume after a bolus had an excellent AUC. The monitor looks promising due to its non-invasive nature and in providing useful information to the clinical in the ED.

Despite advances in the management of pediatric acute respiratory distress syndrome (PARDS) and shock, once refractory to conventional treatment these patients have very high risk of death. Extracorporeal therapies have shown promise in such cases with increased survival rates in refractory shock and PARDS (9). Survival with ECMO in neonates has improved over the decades. However, many of these survivors develop chronic lung disease. In a retrospective series of 91% neonates by Ortiz et al. from a neonatal ECMO centre, the authors report 76% of survivors developing CLD and factors such as prolonged ECMO, early initiation of ECMO (<24 h of life) and Congenital diaphragmatic hernia as risk factors for the same.

Pediatric cardiac critical care one of the most challenging disciplines in medicine has come a long way and in the article by Pollack et al., the authors describe seven key elements including but not limited to education, personalised medicine, newer surgical techniques, nanomedicine, machine learning and quality and safety that will expand the horizon of Pediatric cardiac critical care.

It has been periodically observed that it takes an average of 17 years for adoption of research into practice and this is known as delayed adoption (2). Significant advances have been made in our understanding of the pathophysiology, disease markers and targeted therapies in various critical care conditions and it is continuously evolving. There have been key advances in technology such as use of bed side ultrasound, intra-arterial and intracranial pressure monitoring devices, continuous oximetry, electronic health records and big data. Use of artificial intelligence which is common in surgical conditions is increasingly being used by emergency and critical care specialities and has been reported to be useful in rapid recognition, initiating treatment protocols, continuous monitoring and for prognostication. In this series we have three interesting studies highlighting the use of machine learning, artificial intelligence and big data.

In the first of the three articles published, Kim YT and team attempted to use automated densitometric CT parameters augmented by a machine learning algorithm in 58 children to prognosticate traumatic brain injury in children. About 1/5th of the study population had unfavourable outcome and the prognostic value of brain CT was augmented by the machine learning algorithm. This study provides useful information on use of this algorithm for immediate outcomes during hospitalisation. However, this requires further validation in large scale datasets and whether it has value in predicting long term outcomes also need to be seen.

Abbas et al. similarly compared the utility of a commercially available risk analytic tool T3 (IDO2) in the cardiac intensive care unit with mixed venous saturation (SvO_2_). The AUC for predicting SvO_2_ of <40% was excellent (0.87). Testing the utility of the tool in taking informed clinical decisions and in diverse patient settings would test its true utility and ease of use.

Ehrmann et al. highlight the importance of understanding big data and its interpretation especially by the trainees. While we are moving towards integration of complicated data and developing AI algorithms that would make decision making quick and accurate, the first step towards the clinical utility of such complicated data sets is empowering the trainees with basic understanding of how to use and interpret this data.

Concerted efforts to advance knowledge as well as addressing inequities and resource allocation is needed globally for critically ill children. The insights from our global community is a contribution to this endeavour (10).
